# Prevalence and clinical characteristics of non-malignant CT detected incidental findings in the SUMMIT lung cancer screening cohort

**DOI:** 10.1136/bmjresp-2023-001664

**Published:** 2023-06-15

**Authors:** Sophie Tisi, Andrew W Creamer, Jennifer Dickson, Carolyn Horst, Samantha Quaife, Helen Hall, Priyam Verghese, Kylie Gyertson, Vicky Bowyer, Claire Levermore, Anne-Marie Hacker, Jonathon Teague, Laura Farrelly, Arjun Nair, Anand Devaraj, Allan Hackshaw, John R Hurst, The SUMMIT Consortium, Samuel Janes

**Affiliations:** 1 Lungs for Living Research Centre, UCL Respiratory, University College London, London, UK; 2 Wolfson Institute of Population Health, Queen Mary University of London, London, UK; 3 University College London Hospitals NHS Foundation Trust, London, UK; 4 Cancer Research UK and UCL Cancer Trials Centre, University College London, London, UK; 5 National Heart and Lung Institute, Imperial College London, London, UK; 6 Royal Brompton and Harefield NHS Trust, London, UK; 7 UCL Respiratory, University College London, London, UK

**Keywords:** Lung Cancer, Imaging/CT MRI etc

## Abstract

**Background:**

Pulmonary and extrapulmonary incidental findings are frequently identified on CT scans performed for lung cancer screening. Uncertainty regarding their clinical significance and how and when such findings should be reported back to clinicians and participants persists. We examined the prevalence of non-malignant incidental findings within a lung cancer screening cohort and investigated the morbidity and relevant risk factors associated with incidental findings. We quantified the primary and secondary care referrals generated by our protocol.

**Methods:**

The SUMMIT study (NCT03934866) is a prospective observational cohort study to examine the performance of delivering a low-dose CT (LDCT) screening service to a high-risk population. Spirometry, blood pressure, height/weight and respiratory history were assessed as part of a Lung Health Check. Individuals at high risk of lung cancer were offered an LDCT and returned for two further annual visits. This analysis is a prospective evaluation of the standardised reporting and management protocol for incidental findings developed for the study on the baseline LDCT.

**Results:**

In 11 115 participants included in this analysis, the most common incidental findings were coronary artery calcification (64.2%) and emphysema (33.4%). From our protocolised management approach, the number of participants requiring review for clinically relevant findings in primary care was 1 in 20, and the number potentially requiring review in secondary care was 1 in 25.

**Conclusions:**

Incidental findings are common in lung cancer screening and can be associated with reported symptoms and comorbidities. A standardised reporting protocol allows systematic assessment and standardises onward management.

**WHAT IS ALREADY KNOWN ON THIS TOPIC**

- Incidental findings are common in CT scans performed in lung cancer screening.
- Protocolised approaches to incidental findings have recently been developed, but the outcomes of such approaches have not been reported in prospective large-scale screening programmes.

**WHAT THIS STUDY ADDS**

- Despite incidental findings being identified in over two-thirds of participants, a protocolised approach to non-malignant incidental findings on lung cancer screening CT scans led to review for only 1 in 20 and 1 in 25 participants in primary and secondary care, respectively.

**HOW THIS STUDY MIGHT AFFECT RESEARCH, PRACTICE OR POLICY**

- Protocolised approaches to the reporting of incidental findings in lung cancer screening are essential for the successful implementation of population-level screening in a manner acceptable to participants and demand on downstream primary and secondary care services.

While low-dose CT (LDCT) screening for lung cancer has been demonstrated to reduce lung cancer-associated mortality,^1 2^ uncertainty regarding aspects of screening continues to cause hesitancy to widespread implementation.^3^ While the primary aim of lung cancer screening (LCS) is to identify pulmonary nodules that may represent early lung cancer, LDCT of the thorax may detect other pulmonary and extrapulmonary abnormalities. For some findings such as renal or adrenal nodules, mediastinal masses or breast lumps, the possibility of an extrapulmonary malignancy may be raised, whereas other findings may be clearly non-malignant, but still be potentially associated with morbidity or mortality. Cardiovascular and non-malignant chronic respiratory disease are known to account for substantial mortality and morbidity in LCS cohorts,^1 2 4 5^ and identifying relevant findings on LDCT may offer scope for opportunities to address underdiagnosis with targeted clinical and behavioural intervention.

The reported prevalence of incidental findings at LCS has been estimated to be between 8% and 40%,^6–8^ although published evidence has been confused by varying definitions of what constitutes a ‘clinically relevant’ finding and a lack of standardised reporting of incidentals.^3^ Concerns regarding the financial and workload cost of indiscriminate referrals to primary and secondary care for screen-detected findings have been identified as a potential hurdle to the implementation of population-level screening.^3 9 10^ The importance of developing an approach to managing such findings is heightened following the recent decision by the UK National Screening Committee to support the introduction of LDCT screening for lung cancer in the UK^11^ While the National Health Service (NHS) targeted lung health check (TLHC) has recently published a protocol covering the management of incidental findings,^12^the outcomes in terms of frequency of findings reported and downstream referrals generated to primary and secondary care of such approaches have not been reported in a prospective large scale screening context.

The SUMMIT study (NCT03934866) is an LCS implementation study, where participants at high risk for lung cancer are invited to three annual LHC with LDCT screening. A protocolised approach to management of non-malignant incidental findings was developed based on a systematic review of existing evidence.^13^ Common findings are recorded in a structured manner and a prespecified management approach was developed for each finding.

The aims of this analysis were to (1) examine the prevalence of non-malignant incidental findings within an LCS cohort as assessed by a prespecified, standardised format; (2) explore the characteristics of each incidental finding in terms of association with clinical features and known risk factors for the condition and (3) examine the downstream impact on referrals to primary and secondary care.

## Methods

### Summit study design

The SUMMIT study is a prospective observational cohort study to examine the performance of delivering an LDCT screening service to a high-risk population in London and to validate a multicancer early detection blood test. Consented individuals aged 55–77 at high risk of lung cancer, defined as meeting US Preventative Services Task Force 2013 criteria (at least 30 pack year history and if a former smoker has not given up longer than 15 years ago^14^ or with Prostate Lung Colorectal Ovarian modified 2012 lung cancer risk of ≥1.3%^15^ were offered an LDCT and will return for two further annual visits. This study reports an analysis of all those who attended a baseline study visit from the opening of recruitment in April 2019 to a temporary pause to recruitment in March 2020 following the SARS-CoV-2 pandemic.

### Study procedures

LDCT scans were kept below 2 mSv and were non-ECG gated. Scans were performed in the supine position at maximal inspiration. LDCT scans were reported by thoracic radiologists using a bespoke template allowing categorisation of pulmonary nodules and incidental findings (online supplemental appendix 1).

10.1136/bmjresp-2023-001664.supp1 Supplementary data

Similar to other LCS studies in the UK,^4 16 17^ the SUMMIT screening programme used an LHC model which included spirometry as routine for all participants (unless contraindicated). Prebronchodilator quality-assured spirometry was performed using the Vitalograph Micro spirometer. Subjects prescribed existing bronchodilators were not asked to withhold these. Attendees were encouraged to perform three manoeuvres guided by research practitioners trained to the Association for Respiratory Technology and Physiology standards.^18^ The highest value was recorded with measurements and associated reference values (Global Lung Function Initiative^19^) collected for forced expiratory volume in 1 s (FEV1, forced vital capacity (FVC) and the calculated FEV1/FVC ratio.

A targeted consultation was undertaken to screen for the presence of respiratory symptoms and common respiratory comorbidities (online supplemental appendix 2). Detailed data were collected around lung cancer risk factors. Height, weight and blood pressure were measured. Participants additionally completed an electronic questionnaire which contained questions about general health and lifestyle including any previous exposure to relevant occupational exposures. All current smokers were given Very Brief Advice on smoking cessation and offered referral to local smoking cessation services.

### Incidental findings management protocol

During the development of the SUMMIT Study, a standardised management approach for common incidental findings was developed, which has been previously published.^13^ This protocolised approach to management of non-malignant incidental findings was developed based on a systematic review of existing evidence^13^ and relevant clinical guidelines and sought to deliver a pragmatic, evidence-based approach which was practically deliverable by primary and secondary care. Table 1 is adapted from our previous publication (Horst *et al*^13^) and outlines the assessment criteria and subsequent management actions undertaken for each finding.

**Table 1:** SUMMIT protocolised incidental findings management protocol

Incidental finding and assessment criteria		Onward clinical action
Incidental findings of the pulmonary parenchyma:		
Emphysema		
Qualitative grading by visual assessment of extent of lung affected	Mild (>5%–25%)	Research purposes only
	Moderate (>25%–50%)	
	Severe (>50%–75%)	
	Very severe (>75%)	
Bronchiectasis		
Visual grading based on luminal diameter relative to the accompanying artery diameter	Mild (1.5–2×larger)	Research purposes only
	Moderate (2–3×larger)	
	Severe (>3×larger)	PCP to assess patient and consider referral to secondary care
Interstitial lung abnormality		
Visual assessment of extent of interstitial reticulation and presence or absence of fibrotic features	Mild (<10% reticulation)	Research purposes only
	Moderate (>10% reticulation with no fibrosis)	
	Severe (>10% reticulation with fibrotic features present)	PCP to assess patient and consider referral to secondary care
Cardiovascular incidental findings		
Coronary artery calcification (CAC)		
Each territory (the circumflex, the right coronary artery and the left main plus left anterior descending artery) was assigned a score based on its CAC level: none (0 points), mild (1 point), moderate (2 points) or severe (3 points). Combining the score per territory gave a total overall score out of nine for CAC^44 45^	Mild (1–3)	Research purposes only
	Moderate (4–6)	
	Severe (7–9)	
Thoracic aortic aneurysm		
Calliper measurement of the widest diameter of the ascending thoracic aorta^20^ ^46^ defined as diameter ≥5.0 cm of the ascending aorta or ≥4.0 cm of the descending aorta^46^	4.0–5.5 cm	PCP to refer to secondary care
	>5.5 cm	Direct referral to vascular surgery
Abdominal aortic aneurysm		
Measurement of widest diameter of the abdominal aorta^47^	≥3–5 cm	PCP to refer non-urgently to vascular surgery
	≥ 5 cm	Direct referral to vascular surgery
Aortic valve calcification		
Radiologist assessment of whether the central or peripheral half of valve commissures had calcification present^48^	Central	Research purposes only
	Peripheral	
	Both	
Other incidental findings		
Osteoporotic wedge fracture		
Visual assessment of extent of loss of vertebral height	>50%	PCP to refer for bone density assessment
	<50%	Research purposes only
Pleural findings		
Pleural plaques recorded as present or absent Diffuse pleural thickening refers to a diffuse process with no radiological suspicion of malignancy and was recorded as present or absent (unilateral pleural effusions and unilateral or focal pleural thickening were referred to lung MDT for immediate workup as potential cancer)	Bilateral Pleural Effusions^49^	PCP review
	Diffuse pleural thickening	Annual SUMMIT LDCT
	Pleural plaques	Research purposes only
Hiatus hernia		
	Present	Research purposes only

LDCT, low-dose CT; MDT, multi-disciplinary team; PCP, primary care provider.

### Statistical analysis

The prevalence of incidental findings among the whole cohort on baseline LDCT was assessed, and individual comparative analyses were undertaken for each incidental finding. Differences between groups (presence and absence of the incidental finding) were assessed using the two-sample independent t-test (parametric data) and Mann-Whitney U test (non-parametric data) for continuous variables, and χ^2^ test for categorical data. Statistical significance was defined through p values less than 0.05. Individual univariate and multivariable binary logistic regression analyses were performed to assess the risk of each incidental finding and with adjusted ORs calculated using data on known risk factors associated with that finding. Analysis was performed using SPSS (V.25) and R (V.4.1).

### Public and patient involvement

The protocol, study design and supporting documents for this study underwent review by a participant and public involvement group on several occasions. The invitation materials, participant information sheet, consent form and results letters have been reviewed in detail. Invitation letters were reviewed by patient and public representatives for their readability and acceptability. This was an ongoing process and several of the members of this group continue to be involved by being included on the study steering committee.

## Results

### Baseline characteristics of the cohort

**Figure 1:**
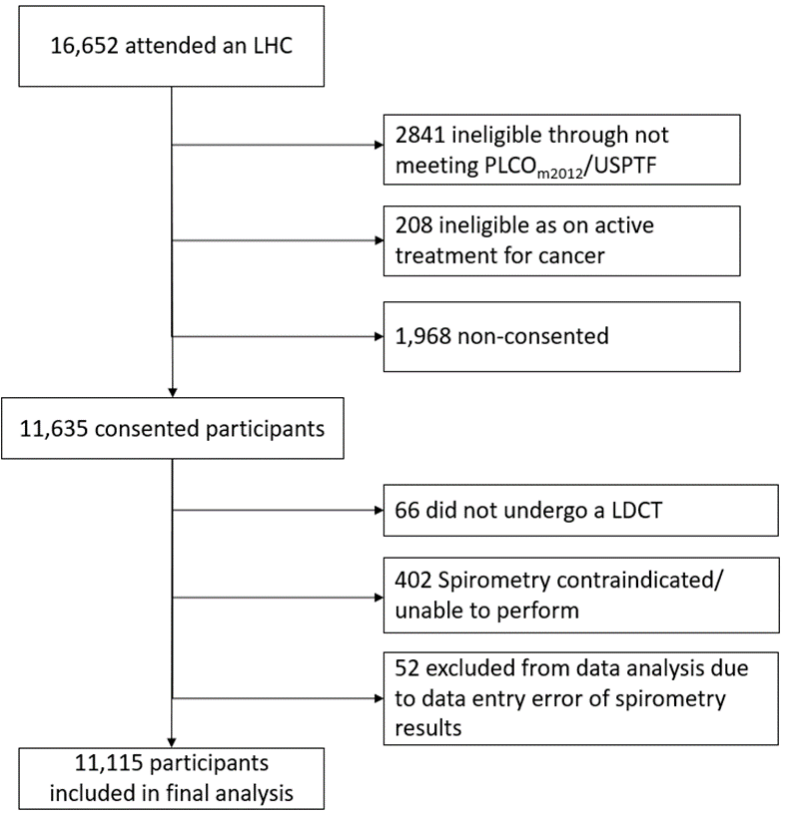
CONSORT (Consolidated Standards of Reporting Trials) diagram. LDCT, low-dose CT; LHC, lung health check; PLCO_m2012_, Prostate Lung Colorectal Ovarian modified 2012; USPSTF, United States Preventive Services Task Force.

Of 16 6652 attendees to an LHC, 13 633 were eligible for inclusion in the study based on predicted lung cancer risk, of which 11 115 consented to baseline LDCT and underwent spirometry and were included in the final analysis (figure 1).

The demographic characteristics of the total cohort (n=11 115) are outlined in table 2. The mean age of the cohort was 65.35 years (SD 6.11), 57.5% were male and 48.6% were current smokers.

**Table 2:** Baseline characteristics of cohort

Characteristic	Value
Age (years)	65.35 (±6.11)
% Male	57.5% (n=6386)
Ethnicity	
White	83.8% (n=9259)
Mixed	2.2% (n=250)
Asian	6.7% (n=743)
Black	4.4% (n=484)
Other	3.4% (n=379)
Education level	
Finished school before 16*	39.5% (n=4389)
High school	23.6% (n=2618)
College	10.9% (n=1216)
Further education	8.4% (n=932)
Bachelors degree	12.6% (n=1296)
Further degree	5.1% (n=564)
IMD quintile	
(Most deprived) 1	31.7% (n=3519)
2	28.7% (n=3190)
3	17.6% (n=1961)
4	15.2% (n=1695)
(Least deprived) 5	5.2% (n=574)
Pack years	45.31 (±23.00)
Current smoker? (yes, %)	48.6% (n=5397)
BMI (kg/m^2^)	28.14 (±9.50)
Systolic BP (mm Hg)	134.08 (±17.61)
Diastolic BP (mm Hg)	79.97 (±10.40)
Airflow obstruction (%)†	49.5% (n=5497)
Personal history of cancer	13.3% (n=1474)
Family history of cancer	19.0% (n=2107)
Median PLCO_m2012_ score	3.08% (1.87%–5.55%)

*In cases where education level was not given this was recorded as ‘finished school before 16’.

†Airflow obstruction defined as prebronchodilator FEV1/FVC ratio of <0.7.

BMI, body mass index; BP, blood pressure; FEV1, forced expiratory volume in 1 s; FVC, forced vital capacity; IMD, index of multiple deprivation; PLCO_m2012_, Prostate Lung Colorectal Ovarian modified 2012.

### Frequency of non-malignant incidental findings

Table 3 summarises the prevalence of individual incidental findings within the total cohort and where appropriate a breakdown of the grade of incidental. The most common radiological findings in the cohort were coronary artery calcification (CAC) (64.2%) and emphysema (33.4%).

**Table 3:** Prevalence of non-malignant incidental findings on baseline LDCT

Incidental finding	LDCT presence (n and % of total cohort)
Emphysema	
TOTAL	3711 (33.4)
Mild	2423 (21.8)
Moderate	900 (8.1)
Severe	331 (3.0)
Very severe	57 (0.5)
Bronchiectasis	
Total	818 (7.4)
Mild	711 (6.4)
Moderate	91 (0.8)
Severe	16 (0.14)
Interstitial lung abnormality	
Total	528 (4.8)
Mild	354 (3.2)
Moderate	62 (0.6)
Severe	112 (1.0)
Coronary artery calcification	
Total	7141 (64.2)
Mild	4035 (36.3)
Moderate	2049 (18.4)
Severe	1057 (9.5)
Thoracic aortic aneurysm	
Total	306 (2.8)
4.0–5.5 cm	301 (2.7)
>5.5 cm	5 (0.1)
Abdominal aortic aneurysm	
Total	2
≥3–5 cm	1
>5 cm	1
Aortic valve calcification	
Total	1808 (16.3)
Central	409 (3.7)
Peripheral	962 (8.7)
Both	437 (3.9)
Osteoporotic wedge fracture	
Total	801 (7.2)
>50%	132 (1.2)
<50%	669 (6.0)
Pleural findings	
Bilateral pleural effusions	7 (0.06)
Diffuse pleural thickening	92 (0.82)
Pleural plaques	599 (5.4)
Hiatus hernia	
Present	1064 (9.6)

LDCT, low-dose CT.

### Emphysema

Emphysema was present in 33.4% (n=3711) of the total cohort; of which in the majority (65.3%, n=2423) was mild (affecting <25% of the total lung). Participants with emphysema had a higher respiratory symptom burden, being more likely to report persistent cough (28.3% vs 21.9%, p<0.001), sputum production (19.6% vs 13.7%, p<0.001), breathlessness (Medical Research Council (MRC) dyspnoea score >1 70.5% vs 63.9%, p<0.001) and respiratory infection frequency (≥2 exacerbations/year 10.1% vs 6.5%, p<0.001, than those without emphysema, online supplemental table A).

While the prevalence of emphysema was higher in those with airflow obstruction than without (67.3% vs 40.1%, p<0.001), 32.3% of participants with radiological emphysema did not have airflow obstruction. Participants with emphysema were more likely to report an existing diagnosis of chronic obstructive pulmonary disease (COPD) (52.3% vs 31.6%, p<0.001), although 47.7% of participants with radiological emphysema did not report a prior diagnosis of COPD.

Multivariate logistic regression analyses demonstrated that increasing age, increasing pack year history and current smoking status were all significantly associated with increased risk of emphysema on baseline LDCT (table 4).

**Table 4:** Emphysema: univariate and multivariate binary logistic regression analyses of factors associated with the presence on baseline LDCT

Variable	Unadjusted OR (95% CI)	P value	Adjusted OR (95% CI)	P value
Age				
Per increasing year	1.037 (1.031 to 1.044)	<0.001	1.025 (1.018 to 1.032)	<0.001
Gender				
Female	1		1	
Male	1.074 (0.991 to 1.163)	0.081	1.051 (0.966 to 1.144)	0.245
Smoking status				
Former smoker	1		1	
Current smoker	1.186 (1.096 to 1.283)	<0.001	1.219 (1.120 to 1.326)	<0.001
Pack year history				
Per increasing pack year	1.007 (1.005 to 1.009)	<0.001	1.005 (1.003 to 1.007)	<0.001
Airflow obstruction				
No airflow obstruction	1		1	
Airflow obstruction	3.118 (2.870 to 3.388)	<0.001	2.888 (2.652 to 3.144)	<0.001

LDCT, low-dose CT.

### Bronchiectasis

Bronchiectasis was identified in 7.3% (818) of the total population. 87% of cases were classed as mild (711/818), with 13% (107/818) classed as moderate or severe (severity classifications as defined in table 1). Only 1.7% (n=14) of those with evidence of bronchiectasis on LDCT self-reported a previous diagnosis of bronchiectasis.

Participants with bronchiectasis on LDCT had a higher respiratory symptom burden than those without bronchiectasis, being more likely to report persistent cough (27.6% vs 23.8%, p=0.013) and sputum production (18.8% vs 15.4%, p=0.009, online supplemental table B). Our protocol only reports severe bronchiectasis to primary care providers (PCPs); compared with participants with mild or moderate bronchiectasis this group were more likely to report persistent cough (43.4% vs 27.3%, p=0.24) and sputum production (31.3% vs 18.6% p=0.336), although this did not reach statistical significance.

The association of radiological bronchiectasis and known risk factors were analysed by binary logistic regression analysis (online supplemental table C). Increasing age, increasing number of respiratory infections in the past year, a history of TB and a history of previous pneumonia were all demonstrated to be independent risk factors for bronchiectasis on multivariate analysis.

### Interstitial lung abnormalities

A total of 528 (4.8%) of the total cohort had evidence of interstitial lung abnormality (ILA) on LDCT with only 6 (1.1%) of these participants reporting a previous diagnosis of an interstitial lung disease (ILD). Participants with ILAs were slightly older (67.52±6.03 vs 65.24±65.24, p<0.001) and were more likely to be male (64.6% vs 57.1%, p=0.001) than those without ILAs (online supplemental table D). There was no difference in levels of current smoking or pack year history between those with and without ILAs on LDCT. There was no difference in respiratory symptoms of persistent cough (26.1% vs 23.9%, p=0.248) or breathlessness (MRC score ≥1 67.4% vs 66.0%, p=0.513).

On univariate and multivariate analyses, the presence of ILAs was independently associated with age, male gender (adjusted odds ratio (AdjOR) 1.272, 95% CI 1.047 to 1.546) and occupational asbestos exposure (adjOR 1.293, 95% CI 1.033 to 1.618) (online supplemental table E).

### Other pulmonary incidental findings

Miscellaneous pulmonary incidental findings included suspected mycobacterial infection (tuberculosis or non-tuberculosis mycobacterium in 8 participants (0.07%) and identification of other likely pulmonary conditions in 10 participants (0.09%, including suspected sarcoidosis, pleuroparenchymal fibroelastosis and pulmonary hypertension). Pleural plaques were found in 5.4% (n=599) and diffuse pleural thickening in 0.8% (n=92). Bilateral pleural effusions were found in seven participants (0.06%).

### Coronary artery calcification

CAC was present in 64.2% (n=7141) of the total cohort (online supplemental table F); which was mild in 56.5% (N=4035), moderate in 28.7% (2,049) and severe in 14.8% (N=1057). On multivariate logistic regression analysis, increasing age, body mass index (BMI), pack year history, elevated systolic blood pressure and current smoking were all significantly associated with the presence of CAC (table 5).

**Table 5:** Coronary artery calcification: univariate and multivariate binary logistic analysis assessing the relationship between associated variables and presence on baseline LDCT

Variable	Unadjusted OR (95% CI)	P value	Adjusted OR (95% CI)	P value
BMI				
Per increasing kg/m^2^	1.01 (1.00 to 1.01)	0.051	1.01 (1.00 to 1.01)	0.037
Age				
Per increasing year	1.10 (1.09 to 1.10)	<0.001	1.09 (1.09 to 1.10)	<0.001
Gender				
Female	1		1	
Male	2.46 (2.27 to 2.66)	<0.001	2.57 (2.35 to 2.81)	<0.001
Blood pressure				
Systolic BP	1.01 (1.01 to 1.01)	<0.001	1.01 (1.01 to 1.01)	<0.001
Diastolic BP	0.994 (0.990 to 0.997)	0.001	0.99 (0.98 to 0.99)	<0.001
Smoking status				
Current smoker	1		1	
Former smoker	1.15 (1.07 to 1.24)	<0.001	1.11 (1.01 to 1.20)	0.023
Pack year history				
Per increasing pack year	1.01 (1.01 to 1.01)	<0.001	1.00 (1.00 to 1.01)	0.001
Ethnicity				
Other	1		1	
White	1.37 (1.12 to 1.69)	0.003	1.46 (1.17 to 1.82)	0.001
Mixed	0.97 (0.70 to 1.33)	0.832	1.27 (0.90 to 1.79)	0.182
Asian	2.29 (1.76 to 2.97)	<0.001	1.88 (1.42 to 2.48)	<0.001
Black	0.65 (0.50 to 0.87)	0.002	0.69 (0.51 to 0.92)	0.010
Airflow obstruction				
No airflow obstruction	1		1	
Airflow obstruction	1.29 (1.20 to 1.40)	<0.001	1.07 (0.98 to 1.16)	0.138

BMI, body mass index; BP, blood pressure; LDCT, low-dose CT.

### Aortic aneurysms

Thoracic aortic aneurysms (TAA) were identified in 2.8% (N=306) of participants, of which 301 (2.7%) were 4.0–5.5 cm and 5 (0.04%) were >5 cm in diameter. Participants with aortic aneurysms were more likely to be male (69.9% vs 57.1%, p<0.001) and older (mean age 66.64 years (±9.75) vs 65.32 (±10.0), p<0.001) (online supplemental table G). On logistic regression analysis age, diastolic blood pressure and male gender remained independent risk factors for the presence of TAA on multivariable models (online supplemental table H).

### Other cardiovascular incidental findings

Aortic valve calcification was present in 16.3% (N=1808) of participants. Pericardial effusions >2 cm were found in three participants.

### Vertebral wedge fractures

Osteoporotic wedge fractures were present in 801 (7.2%) of all individuals (online supplemental table I), with 132 (1.2%) measuring greater than 50% loss and 669 (6.0%) measuring less than 50% loss of vertebral height. Known risk factors for osteoporotic fractures were assessed in logistic regression models (online supplemental table J). Increasing age and low BMI remained independent risk factors for the presence of vertebral wedge fractures on multivariate models. Gender, pack year history and personal history of cancer were not found to have a statistically significant association with presence of vertebral fractures. Airflow obstruction was an independent risk factor for the presence of vertebral fracture. (adjusted OR 1.26; 95% CI 1.08 to 1.46, p=0.003).

### Hiatus hernia

Hiatus hernias were identified in 9.6% (n=1064) of the cohort (online supplemental table K). Binary logistic regression analysis demonstrated that increasing age, female gender and airflow obstruction remained independent risk factors on multivariate analysis (online supplemental table L).

### Implications for primary and secondary care

Our referral policy divided actionable incidental findings into three categories: PCP to manage directly, PCP to assess and consider referral to secondary care, and direct referral to relevant secondary care (table 1). Incidental findings identified on LDCT covered by our management protocol generated a total of 139 (1.3%) referrals for PCP assessment (osteoporotic wedge fractures and bilateral pleural effusions) and 430 (3.6%) referrals for PCP assessment and onward referral (302 referrals for TAA (2.7%) and 128 (1.15%) referrals to respiratory clinics for severe bronchiectasis or ILAs). Six (0.05%) referrals were made directly to secondary care for aortic aneurysms above threshold diameter.

This is in addition to the suspected new pulmonary conditions and pericardial effusions outlined above; these findings were highlighted to physicians working on the study who contacted the participant directly to assess clinical context and arrange appropriate secondary care referral.^20^

## Discussion

We report the prevalence of pulmonary, cardiovascular, and other non-malignant incidental findings identified on LDCT in a large LCS cohort as captured by a protocolised approach to identification and management. By interrogating each finding against known risk factors and reported symptoms we provide a clinical context; highlighting associated morbidity and identifying potentially modifiable risk factors. Finally, by having a standardised management protocol, we can quantify the downstream workload created for primary and secondary care, aiming to maximise the benefit of LDCT in LCS. Our results represent the first analysis of outcomes of a prospectively implemented incidental findings protocol in a large, diverse screening cohort. While there are minor divergences between our protocol and that used by the NHS TLHC programme,^12^ the extent of overlap means our results are highly translatable to anticipating primary and secondary care referrals generated by the imminent national screening programme.^11^

### Comparison to other incidental finding management protocols

Since the SUMMIT study started in early 2019, a number of other management protocols for incidental findings have been published, including the NHS England Quality Assurance standards for the TLHC Programme (V.2, 2022)^12^ and the American College of Radiology (ACR) white paper (2021).^21^ These follow a similar approach to the SUMMIT protocol in terms of distinguishing between findings, which require no further assessment, those requiring assessment by primary care and those warranting specialist opinion. In keeping with the different audiences (clinical screening rather than a research cohort and in different healthcare systems), there are a number of minor discrepancies between these protocols and the SUMMIT approach in how individual findings should be acted on. Nevertheless, our approaches are similar enough for our results to be of use in both understanding the prevalence of non-malignant incidental findings in a lung cancer cohort and in predicting the downstream demand on primary and secondary care services such an approach will generate. Our protocolised management for common incidental findings generated referrals to primary care for 1 in 20 participants (5.1%), and to secondary care for 1 in 25 (1.1% for pulmonary and 2.8% for cardiology/vascular). This is in addition to referrals made for spirometry indicative of undiagnosed COPD, the universal advice to assess QRISK2, and uncommon miscellaneous pulmonary and extrathoracic findings. It is also in addition to findings suspicious for an extrapulmonary malignancy (eg, liver, breast or thyroid nodules) which are referred directly to the relevant secondary care team for further investigation; an analysis of referrals and subsequent diagnostic yield is a planned future publication.

### Clinical significance of radiological incidental findings and opportunities for intervention

Both the SUMMIT protocol and the subsequently published guidelines recognise that there is a distinction between clinically significant incidental findings with established management interventions (such as aortic aneurysms, osteoporotic fractures and bronchiectasis), and findings which may have associated morbidity or prognostic implications, but where at present no evidence exists for specific intervention based purely on radiological findings. Our approach is to report findings in the former category back to PCPs to allow appropriate intervention, while systematically recording the latter for future research purposes without burdening the PCP or participant with knowledge of a finding for which consensus on an evidence-based intervention does not exist.

One area of divergence between the SUMMIT protocol and the ACR white paper and the NHS protocol is the presence of emphysema, with both ACR and the NHS TLHC guidelines advising the finding should prompt ‘consideration’ of referral to community teams. The diagnosis of COPD is made on airflow obstruction in the context of an appropriate exposure and symptoms,^22^ with UK guidelines advising that incidental identification of emphysema on imaging should prompt consideration of spirometry.^23^ As spirometry is already part of our LHCs, our protocol was, therefore, not to report emphysema seen on LDCT. While this is in keeping with current UK guidelines and aims to avoid burdening general practitioners and patients with knowledge of a finding for which there is no specific intervention, we acknowledge further research may show benefit in reporting this back. For example, we found that current smoking is a risk factor for the presence of emphysema. Reporting this finding may, therefore, be an opportunity to support those with long-term tobacco dependence with smoking cessation, an approach currently being explored in the Yorkshire Enhanced Stop Smoking trial.^24^ It is widely recognised that there is systematic underdiagnosis of COPD,^25^ and LCS offers an opportunity to improve diagnosis in a population at risk of this condition. In the UK, the LHC model of delivering LCS with routine spirometry performed for all participants is widely used,^16 17^ and is part of the standard protocol for the NHS TLHC programme.^26^ However, we recognise that international approaches to LCS may vary, and therefore, agree that in screening programmes where the LHC does not include spirometry, emphysema should be reported back to the PCP with the suggestion to perform spirometry.

Conversely, as the diagnosis of bronchiectasis is made radiologically,^27 28^ the combination of this finding with associated symptoms reported at LHC is sufficient to suggest this diagnosis. The correlation with both known risk factors^27 28^ and higher rates of symptoms attributable to the condition in our cohort supports that this was a clinically significant finding. With less than 2% of participants with bronchiectasis on LDCT reporting an existing diagnosis, our results suggest significant undiagnosed disease in this cohort, which could be improved by systematic identification at LCS, justifying our approach to reporting this when present. Our approach matches that of the NHS TLHC protocol, although the NHS TLHC protocol prompts consideration of referral if symptomatic and ‘moderate’ bronchiectasis is present, rather than just ‘severe’.

ILAs refer to specific CT findings potentially compatible with ILD identified in patients without clinical suspicion of the disease.^29^ Shared risk factors for ILD and lung cancer make screening cohorts at elevated risk for the condition, and LCS may offer the opportunity for early diagnosis. However, although the presence of ILAs has been demonstrated to be associated with impaired pulmonary function parameters^30^ and increased all cause mortality,^31^ the natural history and optimal management of these findings remains uncertain.^29^ Unlike bronchiectasis and emphysema, we found no difference in respiratory symptoms reported in participants with ILA compared with those without. Our management protocol reflects the increased risk of progression with established fibrosis identified in other studies.^32^ Since SUMMIT started Fleichner society guidelines have been published on diagnosis and investigating ILAs^29^; future screening studies may wish to use these recommendations as a basis for management of ILA identified in screening.

While several studies have identified an association of CAC with all-cause mortality and cardiovascular events,^33 34^ the evidence is still unclear as to what, if any, specific therapies may be of benefit in this cohort beyond general measures to reduce cardiovascular risk.^35 36^ Consistent guidelines are therefore lacking in whether there is any benefit in reporting specific CAC results at LCS.^37^ In line with British^36^ and US guidelines,^38^ SUMMIT radiologists report the presence and severity of CAC. However, we do not feed this back to PCPs or participants; instead, PCPs are informed all participants are likely to be at elevated risk of cardiovascular disease and advised to assess QRISK score.^39^ There are several reasons we adopted this approach. First, use of QRISK score to decide on introduction of statin therapy is advocated by national guidelines.^40^ Second, previous research has shown that the overwhelming majority (93%–98%)^5 41^ of participants in LCS programmes have a QRISK score of ≥10% (the threshold for statin therapy for primary prevention^40^); individualised reporting is therefore arguably superfluous and risks overwhelming PCPs with information. In the ACR guidelines,^20^ the presence of CAC gives a recommendation for PCP evaluation of cardiovascular disease risk; the same action that is advised for all of our participants. However, we recognise that this differs to some US guidelines, where the presence of moderate or severe CAC as an incidental finding warrants initiation of statin therapy.^38^ Further research is needed to understand the precise role incidentally detected CAC may have in decisions to start lipid-lowering therapy.

Although USPSTF guidelines for osteoporosis screening found insufficient evidence to support this in men,^42^ in our population of previous or current smokers there was no difference between genders and risk of vertebral fractures. In the UK, the NICE guidelines advise that all females over 65 and men over 75 should be screened for osteoporosis.^43^ While screening in men under the age of 75 is advocated in the presence of risk factors such as smoking and secondary causes of osteoporosis such as COPD, these associations are often unrecognised and many men in these categories may not be assessed for fragility fractures. Eighty-eight per cent of the men in our population who had a vertebral fracture were under the age of 75 and so would not meet routine criteria for osteoporosis screening. Additionally, 29% of women with vertebral fractures were under the age of 65. Therefore, there may be increased utility in vertebral wedge fracture identification in the LCS population particularly in men who would not be identified via routine national osteoporosis screening.

### Limitations

While self-reported data were collected on the presence of certain respiratory conditions, participants were not asked about the presence of other comorbidities or current medications, and this self-reported data were not validated against participants hospital or primary care records. While this streamlines the time taken for an LHC appointment to be completed, it limits the ability to determine whether the findings identified were truly incidental or already known about. Consideration must also be given to the potential harms of this approach, particularly psychological; anxiety may be provoked by the identification of unexpected findings and the subsequent diagnostic workup that may follow.

We do not at present have data on how many appointments were made or attended, nor any data on subsequent changes in management. One reason for this is that participants were recruited immediately prior to the COVID-19 pandemic and resulting severe disruption to health services. The time to subsequent healthcare appointments and changes in clinical management during the nationwide lockdown of 2020 are, therefore, unlikely to be representative, and we have, therefore, chosen to present the data in terms of referrals generated, rather than those attended. Data from a UK LCS pilot found that following a referral to primary care for a finding identified at LHC, a change in management was made in 22.6% of participants.^9^ However, the proportion of participants who actually attended a primary care appointment following this referral was relatively low, with between 33.3% and 57.1% (depending on the finding in question) not attending. These results, therefore, highlight that consideration needs to be given not only on how incidental findings are fed back to PCPs, but also to the participants themselves.

### Future research and feasibility for population level screening

While the data presented here identifies participants who may have potential to benefit from identification of incidental findings and quantifies the downstream impact on primary and secondary care services, a more fundamental question is whether this leads to any clinical utility. Future work within the SUMMIT Study will enable the collection of longer-term data from primary and secondary care to more accurately assess the downstream clinical impact of our approach. Furthermore, while outside of the scope of this analysis, the impact of identifying potential extrathoracic malignant findings on LDCT screening is an additional important future research outcome from the SUMMIT study.

We acknowledge that since the development of the SUMMIT study protocol guidelines for the management of incidental findings have been developed, which vary between countries based on populations and healthcare systems.^12 21^ Nevertheless, our findings report the first large-scale prospective implementation of such a standardised approach, feasible at a large scale, which highlights clinically significant findings while minimising extraneous information. Our results describe the demands on primary and secondary care generated by such an approach, and as such are translatable in predicting likely demand generated by the imminent UK screening programme. Such approaches are an essential component of successfully implementing population-level screening.

## Data Availability

Relevant individual deidentified participant data (including data dictionaries) will be made available on reasonable request via email to SJ (s.janes@ucl.ac.uk) following confirmation by SJ and the Cancer Research UK and UCL Cancer Trials Centre. Data will be available to share after the publication of the study primary and secondary endpoints.
