# Supplementary material for: Prevalence and clinical characteristics of non-malignant CT detected incidental findings in the SUMMIT lung cancer screening cohort
Source: BMJ Open Respir Res. 2023 Jun 15;10(1):e001664. doi: 10.1136/bmjresp-2023-001664 (PMC10277548; doi:10.1136/bmjresp-2023-001664)
Supplement: Supplementary data [file bmjresp-2023-001664supp001.pdf]

Appendix 1: Radiology Report proforma for Incidental Findings

9	Emphysema extent	None<br>Unclear<br>Trivial (<5%)<br>Mild (5-25%)<br>Moderate (25-50%)<br>Severe (50-75%)<br>Very severe (>75%)
10	Coronary calcium: LMLAD	None<br>Minimum<br>Mild<br>Moderate<br>Severe<br>Not Reported
11	Coronary calcium: CIR	None<br>Minimum<br>Mild<br>Moderate<br>Severe<br>Not Reported
12	Coronary calcium: RCA	None<br>Minimum<br>Mild<br>Moderate<br>Severe<br>Not Reported
14	Family history of lung cancer	Yes<br>No
15	Please select the applicable pulmonary incidental findings for this patient	None [EXCL]<br>Bronchiectasis<br>Interstitial lung disease<br>Pleural plaques<br>Diffuse pleural thickening<br>Pleural effusion
15a	Bronchiectasis	Mild (1.5-2x artery)<br>Moderate (2-3x artery)<br>Severe- (>3x artery AND >1 segment)
15b	Interstitial lung disease	< 10% reticulation<br>>= 10 % reticulation without fibrotic features<br>>=10% reticulation with fibrotic features
15c	Pleural plaques	Yes<br>No
15d	Diffuse pleural thickening	Yes<br>No

15e	Pleural effusion	Unilateral - Right<br>Unilateral - Left<br>Bilateral
16	Please select the applicable mediastinial and neck incidental findings.	None [EXCL]<br>Thoracic aortic dilatation<br>Anterior mediastinal mass<br>Aortic valve calcification<br>Thyroid nodule
16a	Thoracic aortic dilatation	>= 4 cm and <5.5 cm<br>>= 5.5 cm
16b	Anterior mediastinal mass	< 3 cm, no suspicious features at baseline/no growth on serial imaging<br>< 3 cm, suspicious features or growing on serial imaging<br>>= 3 cm
16c	Aortic valve calcification	Central<br>Peripheral<br>Both
16d	Thyroid nodule	Nodule with fine calcification<br>Nodule associated with local lymphadenopathy<br>Both
17	Please select the applicable subdiaphragmatic incidental findings.	None [EXCL]<br>Adrenal opacity<br>Significant abdominal aortic dilatation<br>Hiatus hernia<br>Osteoporotic wedge fracture
17a	Adrenal opacity	1-4 cm or HU > 10<br>>4 cm
17b	Significant abdominal aortic dilatation	>=3 cm and <5 cm<br>>=5 cm
17c	Hiatus hernia	Absent<br>Present
17d	Osteoporotic wedge fracture	< 50%<br>>= 50%
18	Are there any other emergency non-cancerous findings?	Yes<br>No
18a	Describe the other emergency non-cancerous findings	
19	Is there a likely non-pulmonary malignancy (not already captured in the structured report)?	Yes<br>No

20	Enter information for suspicious non-pulmonary lesion(s)<br><br>Site	
20a	Size (in mm)	
20b	MDT recommendation and other comments	
25	Additional comments on the recommendation	

Appendix 2: Lung Health Check questions regarding respiratory symptoms and respiratory co-morbidities

1	Do you currently have a cough?	Yes<br>No
1a	[If 'yes' to Q1]<br><br>When did the cough start?	Within the last 3 weeks<br>3 to 6 weeks ago<br>6 weeks to 6 months ago<br>6 months to 12 months ago<br>12 months to 24 months ago<br>Greater than 24 months ago
1b	[If 'yes' to Q1]<br>When you cough, do you usually cough up phlegm (sputum)?	Yes<br>No
2	[Show if Q1a is not equal to "Within 3 weeks"]<br>Have you noticed any change in your normal chest symptoms during the past 3 weeks?	Yes<br>No
2a	[Show If 'yes' to Q2]<br>Have your symptoms improved or deteriorated?	Improved<br>Deteriorated
2b	[Show If answer 'deteriorated' to Q2a]<br>[Show if Q1a is not equal to "Within 3 weeks"]<br><br>Has the cough worsened in the last three weeks?	Yes<br>No
2c (i)	[Show If answer 'deteriorated' to Q2a or 'within 3 weeks' to Q1a]<br>We would like to ask some more questions about symptoms which may have changed.<br><br>Fever/ sweats	Yes<br>No
2c (ii)	[Show If answer 'deteriorated' to Q2a or 'within 3 weeks' to Q1a]<br><br>Increased phlegm (sputum) production/ change in the colour of phlegm	Yes<br>No

2c (iii)	[Show If answer 'deteriorated' to Q2a or 'within 3 weeks' to Q1a]<br><br>Increased shortness of breath	Yes<br>No
2c (iv)	[Show If answer 'deteriorated' to Q2a or 'within 3 weeks' to Q1a]<br><br>Increased wheeze (noisy breathing)	Yes<br>No
2c (v)	[Show If answer 'deteriorated' to Q2a or 'within 3 weeks' to Q1a]<br><br>Sharp chest pain when you take a deep breath (pleuritic pain)	Yes<br>No
3	Are you currently taking antibiotics or steroids prescribed for an acute chest infection?	Yes<br>No
4	How many times in the past 12 months have you used antibiotics or steroids for your chest?	[number input][range 0:50]
9	Have you coughed up blood in the last year?	Yes<br>No
9a	[Show if yes to Q9]<br>Have you coughed up blood within the past two weeks?	Yes<br>No
9b	[Show if yes to Q9]<br>Has the blood been investigated by a doctor?	Yes<br>No
10	Which of these best describes your breathing?	[Only one option can be selected]<br><br>Only breathless on strenuous exercise<br><br>Breathless when hurrying on the flat or up a slight hill<br><br>Slower than peers when walking. Would need to stop after 15 minutes or 1 mile at own pace<br><br>Would need to stop due to breathlessness after 100 yards on the flat<br><br>Too breathless to leave house or when washing/dressing

		Unable to answer questions as limited due to other co-morbidity
11	Have you lost weight in the past three months?	Yes<br>No
11a	[Show if 'yes' to Q11]<br>Was the weight loss intentional?	Yes<br>No
11b	[Show if 'yes' to Q11]<br>Do you know how much weight you have lost in the past three months?	Yes<br>No
11c	[Show if 'yes' to Q11b]<br>How much weight have you lost?	[Number input]<br>_______Kg<br>or<br>________Ib
11d	[Show if 'no' to Q11a]<br>Has this unintentional weight loss been investigated by a doctor?	Yes<br>No
	Medical History	
12	Have you ever been told you have any of the following conditions?	
12i	COPD/chronic bronchitis/emphysema	Yes<br>No<br>[Field to auto populate from phone screener Q6, with ability to edit response at LHC]
12ii	Asthma	Yes<br>No
12iii	Atopy - hayfever/eczema/rhinitis	Yes<br>No
12iv	Pulmonary fibrosis	Yes<br>No
12v	Bronchiectasis	Yes<br>No
12vi	Previous Pneumonia	Yes<br>No
12vii	Sarcoidosis	Yes<br>No
12viii	Tuberculosis (TB)	Yes<br>No
12viii a	[show if 'yes' to Q12viii]<br>Did or do you have pulmonary or non-pulmonary Tuberculosis (TB)?	Lung only (Pulmonary)<br>Outside the lung only (extra-pulmonary)<br>Lung and elsewhere in the body<br>I don't know
12viii b	[show if 'yes' to Q12viii]<br>Are you currently receiving treatment for Tuberculosis (TB)?	Yes<br>No

	Family History	
14	Have your parents, brother, sister, or children ever been diagnosed with lung cancer?	Yes<br>No<br><br>[Field to auto populate from phone screener Q8, with ability to edit response at LHC]
	Demographics	
15	Which of these categories best describes your ethnic group?	White British<br>White Irish<br>Other White<br>White and Black Caribbean<br>White and Black African<br>White and Asian<br>Chinese<br>Other Asian<br>Black Caribbean<br>Black African<br>Other Black<br>Indian<br>Pakistani<br>Bangladeshi<br>Other Mixed<br>Any other ethnic group
16	What is the highest level of education you have achieved?	Finished school at or before the age of fifteen<br>Completed CSEs, O-levels or equivalent<br>Completed A-levels or equivalent<br>Completed further education but not a degree<br>Completed a Bachelor’s degree or equivalent<br>Completed a further degree e.g. masters or PhD etc<br><br>[Field to auto populate from phone screener Q5, with ability to edit response at LHC]
	Smoking History	
17	Have you smoked more than 100 cigarettes in your lifetime?	Yes<br>No<br>[Field to auto populate from phone screener Q1, with ability to edit response at LHC]
17a	[If yes to Q17]<br>Do you currently smoke cigarettes regularly?	Yes<br>No

		[Field to auto populate from phone screener Q3, with ability to edit response at LHC]
17b	At what age did you start smoking cigarettes regularly?	_ years<br><br>[Field to auto populate from phone screener Q3c, with ability to edit response at LHC] [range 1:78]
17c	[If no to Q17a]<br>At what age did you stop smoking cigarettes regularly?	[number input]<br>_ Years<br><br>[Field to auto populate from phone screener Q3b, with ability to edit response at LHC] [range 1:78]
17d	[If yes to Q17a]<br>During the time you have smoked cigarettes, have you ever stopped smoking for more than one month?	Yes<br>No<br><br>[Field to auto populate from phone screener Q3d, with ability to edit response at LHC]
17e	[If yes to 17d]<br>How many months did you stop for in total?<br>[Range 1-500]	Months<br><br>[Field to auto populate from phone screener Q3d(i), with ability to edit response at LHC]
17f	How many cigarettes do or did you smoke per day on average for the majority of your time as a smoker?	__ number of cigarettes per day<br><br>or<br>__ grams of tobacco per week<br><br>[Field to auto populate from phone screener Q3e or Q3f, with ability to edit response at LHC]
18	Have you ever smoked any of the following types of tobacco in addition to or instead of cigarettes?	[Please select all that apply]<br>Cigars<br>Cigarillos<br>Pipe<br>Marijuana<br>Waterpipe<br>None of the above
18a	[show if selects cigars in Q18]<br>How often do or did you smoke cigars?	[please select one option]<br><br>Occasionally (less than weekly)<br>Regularly (at least once per week)
18b	[Show if select regularly to Q18a]<br>Do you smoke cigars currently?	Yes<br>No
18c	[Show if select regularly to Q18a]<br>At what age did you start smoking cigars?	[number input]

18d	[Show if select regularly to Q18a and no to Q18b]<br>At what age did you stop smoking cigars?	[number input]
18e	[Show if select regularly to Q18a]<br>How many cigars do or did you smoke per week on average for the majority of your time as a smoker?	[number input]<br>_ per week
18f	[show if selects cigarillos in Q18]<br>How often do or did you smoke cigarillos?	Occasionally (less than weekly)<br>Regularly (at least once per week)
18g	[Show if select regularly to Q18f]<br>Do you smoke cigarillos currently?	Yes<br>No
18h	[Show if select regularly to Q18f]<br>At what age did you start smoking cigarillos?	[number input]
18i	[Show if select regularly to Q18f and no to Q18g]<br>At what age did you stop smoking cigarillos?	[number input]
18j	[Show if select regularly to Q18f]<br>How many cigarillos do or did you smoke per week on average for the majority of your time as a smoker?	[number input]<br>per week
18k	[show if selects pipe in Q18]<br>How often do or did you smoke a pipe?	Occasionally (less than weekly)<br>Regularly (at least once per week)
18l	[Show if select regularly to Q18k]<br>Do you smoke a pipe currently?	Yes<br>No
18m	[Show if select regularly to Q18k]<br>At what age did you start smoking a pipe?	[number input]
18n	[Show if select regularly to Q18k and no to Q18l]<br>At what age did you stop smoking a pipe?	[number input]
18o	[Show if select regularly to Q18k]<br>How many pipe bowls do or did you smoke per week on average for the majority of your time as a smoker?	[number input]<br>per week
18p	[show if selects marijuana in Q18]<br>How often do or did you smoke marijuana?	Occasionally (less than weekly)<br>Regularly (at least once per week)<br>Decline to answer
18q	[Show if select regularly to Q18p]<br>Do you smoke marijuana currently?	Yes<br>No
18r	[Show if select regularly to Q18p]<br>At what age did you start smoking marijuana?	[number input]
18s	[Show if select regularly to Q18p and no to Q18q]<br>At what age did you stop smoking marijuana?	[number input]
18t	[Show if select regularly to Q18p]	[number input]<br>per week

	How many joints of marijuana do or did you smoke per week on average for the majority of your time as a smoker?	
18u	[show if selects waterpipe in Q18]<br>How often do or did you use a waterpipe (20 minute session)?	Occasionally (less than weekly)<br>Regularly (at least once per week)
18v	[Show if select regularly to Q18u]<br>Do you smoke a waterpipe currently?	Yes<br>No
18w	[Show if select regularly to Q18u]<br>At what age did you start smoking a waterpipe?	[number input]
18x	[Show if select regularly to Q18u and no to Q18v]<br>At what age did you stop smoking a waterpipe?	[number input]
18y	[Show if select regularly to Q18u]<br>For how many sessions (20 minutes) do or did you use a waterpipe per week on average for the majority of your time as a smoker?	[number input]<br>per week
	Smoking cessation	
19	[Show if yes to Q17a or 18b or 18g or 18l]<br>Please confirm that Very Brief Advice (VBA) on smoking cessation has been given	Yes<br>No
19a	[Show if 'No' to Q19]<br>If no VBA given, please briefly explain why	[Free text]
20	[if yes to Q17a or 18b or 18g or 18l]<br>Has the participant consented to a smoking cessation referral being made on their behalf?<br>This includes consent to their information being shared with a stop smoking service and to being contacted about the referral by that service.	Yes<br>No, the participant would prefer to self-refer<br>No, the participant does not want support from a stop smoking service<br>No, already in contact with a stop smoking service
	Clinical recordings	
23	Height<br>[range:60-280]	__cm<br>[numerical input to 1.dp]
24	Weight<br>[range:25-350]	__ kg<br>[numerical input to 1.dp]
25	Has the participant had their blood pressure taken?	Yes<br>Declined
25a	[Show if yes to Q25]<br>BP (systolic)<br>[range:30-250]	__ mmHg

25b	[Show if yes to Q25]<br>BP (diastolic)<br>[range:10-250]	__ mmHg
26a	Was spirometry cancelled due to Covid-19 related social distancing guidelines?	Yes<br>No
26b	[show if "No" to 26a]<br>Is spirometry contraindicated?	Yes<br>No
26c	[Show if no to Q26b]<br>FEV1<br><br>[Range 0.10L to 9.99L]	__ litres<br>[numerical input to 2. dp]
26d	[Show if no to Q26b]<br>FEV1 % Predicted<br><br>[Max 250%]	__ %<br>[numerical to 1.dp]
26e	[Show if no to Q26b]<br>FVC<br><br>[Range 0.1L to 9.99L]	__ litres<br>[numerical input to 2 dp]
26f	[Show if no to Q26b]<br>FVC % Predicted<br><br>[Max 250%]	__ %
26g	[Show if no to Q26b]<br>FEV1: FVC<br>[Range 0-1]	[Numerical input to 2.dp]
27	BMI<br>[This should be calculated, no need for staff to enter]<br>[Hidden from the nurse/UI]	__ kg/m2
28	USPSTF criteria met?<br>[This should be calculated, no need for staff to enter]<br>[Hidden from Nurse/UI]	Yes<br>No
29	PLCO risk score<br>[This should be calculated, no need for staff to enter]<br>[Hidden from Nurse/UI]	Numeric
30	Smoking pack years<br>[This should be calculated, no need for staff to enter]<br>[Hidden from Nurse/UI]	Numeric

Appendix 3: Supplementary data

	Emphysema (n=3711)	No emphysema (n=7404)	p value
**Age (years)**	66.26 (±6.01)	64.90 (±6.11)	<0.001
**Male (%)**	58.6% (n=2175)	56.9% (n=4211)	0.081
**Current smoker (%)**	51.4% (n=1907)	47.1% (n=3490)	<0.001

**Pack year history (years)**	47.84 (±22.72)	44.05 (±23.03)	<0.001
**Airflow obstruction? (%)**	67.8% (n=2515)	40.3% (n=2982)	<0.001
**Cough > 6 weeks (%)**	28.3% (n=1052)	21.9% (n=1620)	<0.001
**Sputum (%)**	19.6% (n=727)	13.7% (n=1012)	<0.001
**MRC score ≥1 (%)**	70.5% (n=2618)	63.9% (n=4730)	<0.001
**≥2 exacerbations per year (%)**	10.1% (n=375)	6.5% (n=479)	<0.001
**Self-reported diagnosis of COPD**	52.3% (n=2126)	31.6% (n=2342)	<0.001

Supplementary Table A: Characteristics of those with emphysema on baseline LDCT compared to those without

	**Bronchiectasis (n=818)**	**No bronchiectasis (n=10,297)**	**p value**
**Age (years)**	67.33 (±5.88)	65.20 (±6.10)	<0.001
**Male? (%)**	59.8% (n=489)	57.3% (n=5897)	0.162
**Cough > 6 weeks (%)**	27.6% (n=226)	23.8% (n=2448)	0.013
**Sputum (%)**	18.8% (n=154)	15.4% (n=1585)	0.009
**MRC score ≥1 (%)**	67.8% (n=555)	66.0% (n=6793)	0.275
**Haemoptysis in past year? (%)**	2.6% (n=21)	2.3% (n=237)	0.627
**Exacerbations in past year (median)**	0.00 (0.00-1.00)	0.00 (0.00-0.00)	<0.001
**≥2 exacerbations per year (%)**	10.1% (n=83)	7.5% (n=771)	0.006
**Previous pneumonia (%)**	20.8% (n=170)	14.5% (n=1495)	<0.001
**Previous TB (%)**	4.4% (n=36)	1.9% (n=199)	<0.001
**Self-reported bronchiectasis (%)**	1.7% (n=14)	0.7% (n=75)	0.002
**FEV1% predicted (%)**	74.01 (±22.14)	76.16 (±19.58)	0.003
**Airflow obstruction (%)**	54.5% (n=446)	49.1% (n=5051)	0.003

Supplementary Table B: Characteristics of those with and without bronchiectasis on baseline LDCT

Variable	Unadjusted OR (95% CI)	p	Adjusted OR (95% CI)	p
**Age**				
Per increasing year	1.06 (1.05-1.07)	<0.001	1.05 (1.04-1.07)	<0.001
**Gender**				

Male	1		1	
Female	0.902 (0.780-1.043)	0.162	0.87 (0.75-1.01)	0.064
**Previous TB**				
No history of TB	1		1	
Previous TB	2.332 (1.626-3.356)	<0.001	2.20 (1.53-3.12)	<0.001
**Pneumonia**				
No previous history	1		1	
History of pneumonia	1.545 (1.293-1.845)	<0.001	1.42 (1.19-1.71)	<0.001
**Smoking status**				
Current smoker	1	<0.001	1	
Former smoker	1.49 (1.29-1.72)		1.22 (1.45-1.54)	<0.001
**Exacerbations in past 12 months**				
Per increasing exacerbation	1.12 (1.06-1.12)	<0.001	1.10 (1.04-1.17)	0.002
**Airflow obstruction**				
No airflow obstruction	1		1	
Airflow obstruction	1.25 (1.08-1.44)	0.003	1.08 (0.93-1.25)	0.305

Supplementary Table C: Bronchiectasis: Univariate and multivariate binary logistic analysis assessing the relationship between associated variables and presence on baseline LDCT

	ILD (n=528)	No ILD (n= 10,587)	p value
**Age (years)**	67.52 (±6.03)	65.24 (±6.09)	<0.001
**Male Sex (%)**	64.6% (n=341)	57.1% (n=6045)	0.001
**Current smoker (%)**	47.5% (n=251)	48.6% (n=5146)	0.631
**Pack year history (years)**	45.80 (±25.70)	45.29 (±22.86)	0.620
**Cough > 6 weeks (%)**	26.1% (n=138)	23.9% (n=2534)	0.248
**MRC score ≥1 (%)**	67.4% (n=356)	66.0% (n=6992)	0.513
**Self-reported ILD (%)**	1.1% (n=6)	0.3% (n=37)	0.004
**FEV1% predicted (%)**	77.98 (±18.60)	75.91 (±19.84)	0.019
**Emphysema present (%)**	37.3% (n=197)	33.2% (n=3541)	0.050
**Airflow obstruction (%)**	42.8% (n=226)	49.8% (n=5271)	0.002

Supplementary Table D: Characteristics of those with and without ILA on baseline LDCT

Variable	Unadjusted OR (95% CI)	p	Adjusted OR (95%CI)	p
**Age**				
Per increasing year	1.062 (1.049-1.079)	<0.001	1.072 (1.055-1.088)	<0.001
**Gender**				
Female	1		1	
Male	1.370 (1.142-1.644)	0.001	1.272 (1.047-1.546)	0.016
**Pneumonia**				

No previous history	1		1	
History of pneumonia	0.952 (0.742-1.221)	0.699	0.942 (0.731-1.214)	0.646
**Smoking status**				
Current smoker	1		1	
Former smoker	1.044 (0.876-1.243)	0.632	0.878 (0.733-1.052)	0.159
**Pack years**				
Per increasing year	1.001 (0.997-1.005)	0.620	1.00 (0.996-1.003)	0.852
**Occupational Asbestos exposure**				
**No**	1		1	
**Yes**	1.389 (1.127-1.712)	0.002	1.293 (1.033-1.618)	0.025
**Airflow obstruction**				
No airflow obstruction	1		1	
Airflow obstruction	0.755 (0.633-0.990)	0.002	0.608 (0.504-0.734)	<0.001

Supplementary Table E: Interstitial Lung Abnormalities: Univariate and multivariate binary logistic analysis assessing the relationship between associated variables and presence on baseline LDCT

	CAC present (n=7141)	No CAC (n=3974)	p value
**Age (years)**	66.52 (±5.98)	63.26 (±5.78)	<0.001
**Male Sex(%)**	65.3% (n=4663)	43.4% (n=1723)	<0.001
**BMI (kg/m^2)**	28.28 (±9.63)	27.90 (±9.24)	0.040
**Systolic BP (mmHg)**	135.34 (±17.68)	131.83 (±17.23)	<0.001
**Systolic BP ≥140mmHg (%)**	40.7% (n=2906)	32.1% (n=1275)	<0.001
**Systolic BP ≥160mmHg (%)**	8.4% (n=598)	5.9% (n=233)	<0.001
**Diastolic BP (mmHg)**	79.72 (±10.53)	80.41 (±10.15)	0.001
**Current smoker (%)**	47.3% (n=3378)	50.8% (n=2019)	<0.001
**Pack year history (years)**	46.67 (±24.19)	42.88 (±20.48)	<0.001
**Ethnicity**			
**White**	84.0% (n=5997)	82.1% (n=3262)	<0.001
**Mixed**	2.0% (n=141)	2.7% (n=109)	
**Asian**	7.8% (n=560)	4.6% (n=183)	
**Black**	3.2% (n=226)	6.5% (n=258)	
**Other**	3.0% (n=217)	4.1% (n=162)	
**Airflow obstruction (%)**	51.8% (n=3696)	45.3% (n=1801)	<0.001

Supplementary Table F. Baseline characteristics of those with CAC compared to those without CAC on baseline LDCT

	Thoracic aneurysm present (N=306)	No aneurysm present (N=10809)	p value
**Age (years)**	66.64 (±9.75)	65.32(±10.00)	<0.001
**Male Sex(%)**	69.9% (n=214)	57.1%(N=6172)	<0.001
**BMI (kg/m²)**	28.08 (±5.90)	28.15 (±6.61)	0.810
**Systolic BP (mmHg)**	135.5 (±22.0)	134.0 (±24.0)	<0.001
**Systolic BP ≥140mmHg (%)**	38.9% (n=119)	37.6% (n=4062)	0.685
**Systolic BP ≥160mmHg (%)**	9.5% (n=29)	7.4% (n=802)	0.215
**Diastolic BP (mmHg)**	83.2 (IQR±14)	79.88 (±14)	<0.001
**Current smoker (%)**	47.3% (n=3378)	50.8% (n=2019)	<0.001
**Pack year history (years)**	43.46 (±19.25)	45.36 (±18.75)	0.136
**Ethnicity**			
**White**	84.0% (n=5997)	82.1% (n=3262)	<0.001
**Mixed**	2.0% (n=141)	2.7% (n=109)	
**Asian**	7.8% (n=560)	4.6% (n=183)	
**Black**	3.2% (n=226)	6.5% (n=258)	
**Other**	3.0% (n=217)	4.1% (n=162)	
**Airflow obstruction (%)**	50.7% (n=155)	49.4% (n=5342)	0.714

Supplementary Table G: Baseline characteristics of those with and without thoracic aortic aneurysm on baseline LDCT

Variable	Unadjusted OR (95% CI)	p	Adjusted OR (95% CI)	p
**BMI**				
Per increasing kg/m²	0.998 (0.980-1.008)	0.822	0.998 (0.980-1.007)	0.827
**Age**				

Per increasing year	1.036 (1.017 – 1.055)	<0.001	1.048 (1.027- 1.069)	<0.001
**Gender**				
Female	1		1	
Male	1.748 (1.370 – 2.248)	<0.001	1.655 (1.290 – 2.139)	<0.001
**Blood pressure**				
Systolic BP	1.010 (1.004-1.017)	0.001	0.998 (0.990 – 1.005)	0.537
Diastolic BP	1.030 (1.019 – 1.040)	<0.001	1.032 (1.020 – 1.045)	<0.001
**Smoking status**				
Former smoker	1		1	
Current smoker	1.019 (0.812-1.279)	0.869	1.126 (0.890 – 1.425)	0.321
**Pack year history**				
Per increasing pack year	0.996 (0.990 – 1.001)	0.152	0.994 (0.988 – 1.000)	0.044
**Airflow obstruction**				
No airflow obstruction	1		1	
Airflow obstruction	1.051 (0.837 – 1.319)	0.671	1.000 (0.790-1.263)	0.990

Supplementary Table H: Thoracic Aortic Aneurysm: Univariate and multivariate binary logistic analysis assessing the relationship between associated variables and presence on baseline LDCT

	**Vertebral wedge fracture *(n=801)***	**No vertebral wedge fractures *(n=10,314)***	**p value**
**Prevalence in males**	467/6386 (7.3%)	-	
**Prevalence in females**	334/4729 (7.1%)	-	
**Age (years)**	67.24 (±6.15)	65.21 (±6.08)	<0.001
**Female (%)**	41.7 % (n=334)	42.6% (n=4395)	0.614
**BMI (kg/m^2)**	27.42 (±5.24)	28.20 (±9.74)	0.026
**Pack year history**	45.98 (±22.36)	45.26 (±23.05)	0.394
**Current smoker (%)**	47.7% (n=382)	48.6% (n=5015)	0.611
**Personal history of cancer (%)**	15.4% (n=123)	13.1% (n=1351)	0.07
**IMD rank**	12,147.84 (±7660.29)	12068.96 (±7797.61)	0.784
**Exacerbations over last year**	0.00 (0.00-0.00)	0.00 (0.00-0.00)	0.351
**Airflow obstruction (%)**	58.1% (n=465)	48.8% (n=5032)	<0.001

Supplementary Table I: Characteristics of those with and without the presence of vertebral wedge fractures on baseline LDCT

Variable	Unadjusted OR	p	Adjusted OR	p
**BMI**				
Per increasing kg/m^2	0.98 (0.97-0.99)	0.004	0.99 (0.97-1.00)	0.043
**Age**				
Per increasing year	1.06 (1.04-1.07)	<0.001	1.05 (1.04-1.07)	<0.001
**Gender**				
Male	1		1	
Female	0.96 (0.83-1.11)	0.61	0.95 (082-1.10)	0.505
**Smoking status**				
Current smoker	1		1	
Former smoker	1.04 (0.90-1.20)	0.611	0.96 (0.82-1.11)	0.957

**Pack year history**				
Per increasing pack year	1.00 (1.00-1.00)	0.39	1.00 (1.00-1.00)	0.885
**Personal history of cancer**				
No	1		1	
Yes	1.20 (0.98-1.47)	0.07	1.1 (0.90-1.35)	0.368
**Airflow obstruction**				
No airflow obstruction	1		1	
Airflow obstruction	1.45 (1.26-1.68)	<0.001	1.26 (1.08-1.46)	0.003

Supplementary Table J: Vertebral wedge fractures: Univariate and multivariate binary logistic analysis assessing the relationship between associated variables and presence on baseline LDCT

	**Hiatus Hernia** ***(n=1068)***	**No hiatus hernia** ***(n=10,047)***	**p value**
**Age (years)**	67.59 (±5.92)	65.12 (±6.08)	<0.001
**Male (%)**	49.3% (n=527)	58.3% (n=5859)	<0.001
**BMI (kg/m^2)**	28.69 (±5.20)	28.08 (±9.84)	0.046
**Current smoker (%)**	38.5% (n=411)	49.6% (n=4986)	<0.001
**Airflow obstruction (%)**	55.9% (n=597)	48.85 (n=4900)	<0.001

Supplementary Table K: Characteristics of participants with and without the presence of a hiatus hernia on baseline LDCT

**Variable**	**Unadjusted OR (95% CI)**	**p**	**Adjusted OR (95%CI)**	**p**
**Age**				
Per increasing year	1.069 (1.058-1.080)	<0.001	1.062 (1.050-1.073)	<0.001
**Gender**				
Female	1		1	
Male	0.696 (0.614-0.790)	<0.001	0.693 (0.610-0.788)	<0.001
**BMI**				
Per increasing kg/m^2	1.004 (1.000-1.009)	0.061	1.005 (1.000-1.009)	0.059
**Smoking status**				
Current smoker	1		1	
Former smoker	1.575 (1.384-1.792)	<0.001	1.379 (1.207-1.575)	<0.001
**Airflow obstruction**				
No	1		1	
Yes	1.331 (1.173-1.512)	<0.001	1.195 (1.049-1.362)	0.007

Supplementary Table L: Hiatus Hernia: Univariate and multivariate binary logistic analysis assessing the relationship between associated variables and presence on baseline LDCT
